
JOURNAL INFORMATION
==============================
NLM Title Abbreviation: ScientificWorldJournal
Journal ID: ScientificWorldJournal
NLM Journal ID: 101131163
Journal ID: TSWJ

The Scientific World Journal

ISSN: 2356-6140
EISSN: 1537-744X
Publisher: Wiley

ARTICLE INFORMATION
==============================
PMCID: PMC6084738
PMID: 30147549
DOI: 10.1100/tsw.2001.139
Article ID: 843515
Article version: 1
Subjects: Short Report

SIR2 Proteins Regulate Aging in Response to Nutrients

Guarente Leonard *
Department of Biology, MIT, Cambridge, MA 02139, USA
*Leonard Guarente: leng@mit.edu
Publication date: 2001
Electronic publication date: 2001 Dec 19
Volume: 1
First page: 75
Last page: 75
Copyright: Copyright © 2001 Leonard Guarente.
Copyright year: 2001
License: This is an open access article distributed under the Creative Commons Attribution License, which permits unrestricted use, distribution, and reproduction in any medium, provided the original work is properly cited.
License URL: https://creativecommons.org/licenses/by/3.0/

==============================
Miami *Nature Biotechnology* Short Reports
*TheScientificWorld* (2001) 1(S3), 75SR
ISSN 1532-2246; DOI 10.1100/TSW.2001.139

### SIR2 PROTEINS REGULATE AGING IN RESPONSE TO NUTRIENTS

Leonard Guarente

Department of Biology, MIT, Cambridge, MA 02139
leng@mit.edu

Studies on the molecular basis of aging in my lab over the past nine years have led to the notion that the SIR2 gene controls the pace of aging in response to nutrient availability. SIR2 promotes longevity: sir2 deletion strains of yeast have short life spans, and strains with an extra copy of SIR2 live longer than wild type. Sir2p triggers silencing at select genomic locations, including mating type genes, telomeres, and the ribosomal DNA (rDNA). Silencing at this last locus represses the formation of toxic rDNA circles by recombination and thus potentiates a long life span.

Sir2p was shown to be an NAD-dependent histone deacetylase (see figure below). This finding explains how Sir2p creates silenced chromatin and, surprisingly, draws a link between metabolic rate and aging. We suggest that as metabolic rate slows, for example during calorie restriction, NAD is freed up from its many metabolic reactions and activates Sir2p. This promotes an increase in silencing and a longer life span. We show experimentally that SIR2 and NAD are involved in the extention of yeast life span by calorie restriction. We imagine that this mechanism has been selected because it allows organisms to stay alive longer in the face of adversity, to resume reproduction when conditions improve. It is interesting to note, in this regard, that SIR2 also controls mating and thus sporulation, a pathway of cell differentiation that allows long-term survival in the face of scarcity.

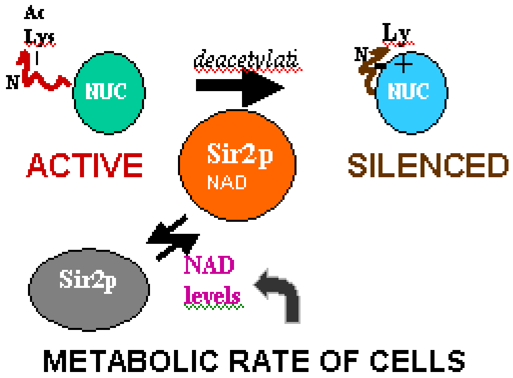

More recently we have begun experiments to determine whether SIR2 gene homologs also regulate aging in animals. Two systems have been used, C. elegans and mice. Studies in C. elegans that relate sir-2 genes to aging in that organism will be described. Models will be put forward to suggest how Sir2 proteins could be general regulators of aging.

**REFERENCES.**

1. Kaeberlein, M., McVey, M., and Guarente, L. (1999) Genes Dev. 13, 2570-2581
2. Guarente, L. (1999) Nat. Genet. 23, 281-286
3. Guarente, L. (1999) Nature 402, 243-245
4. Imai, S., Armstrong, C., and Guarente, L. (2000) Nature, 403, 795-800
5. Lin, S.J., Defossez, P.-A., and Guarente, L. (2000) Science 289, 2126-2128
